# Fracture Toughness Estimation of Single-Crystal Aluminum at Nanoscale

**DOI:** 10.3390/nano11030680

**Published:** 2021-03-09

**Authors:** Wilmer Velilla-Díaz, Luis Ricardo, Argemiro Palencia, Habib R. Zambrano

**Affiliations:** 1 Departamento de Ingeniería Mecánica, Universidad Autónoma del Caribe, Barranquilla 080020, Colombia; luis.ricardo76@uac.edu.co (L.R.); Argemiro.palencia15@uac.edu.co (A.P.); 2 Departamento de Ingeniería Mecánica, Universidad del Norte, Barranquilla 081007, Colombia; hzambrano@uninorte.edu.co

**Keywords:** fracture toughness, molecular dynamics, single-crystals, crack tip opening displacement, aluminum

## Abstract

In this publication, molecular dynamics simulations are used to investigate the fracture behavior of single-crystal aluminum. The stress intensity factor is estimated by means of four different methods, the accuracy is assessed for each approach and the fracture toughness is estimated. The proposed methodology is also applied to estimate the fracture toughness for graphene and diamond using published data from other scientific articles. The obtained fracture toughness for the single-crystal aluminum is compared with other nanomaterials that have similar microstructures. Dislocation emission during the fracture simulation of the cracked nano-crystal of aluminum is analyzed to study the fracture behavior. Brittle fracture behavior is the predominant failure mode for the nanomaterials studied in this research.

## 1. Introduction

Molecular dynamics (MD) simulations are successfully utilized to investigate tensile properties of nano-crystals such as ultimate tensile strength ($S_{U}$) and elastic modulus (*E*) [1,2,3,4,5,6]. Recently, cracked nano-crystals have been modeled by means of MD to investigate the fracture mechanics (FM) properties, which have been estimated for different nano-crystalline materials [7,8,9,10,11,12]. At the nanoscale, different methodologies have been proposed to evaluate fracture toughness by means of MD simulations of cracked nano-crystals [13,14,15,16,17,18,19,20,21,22,23,24]. Fracture toughness is the FM property that quantifies the material resistance to unstable crack propagation, which is usually obtained by means of the Energy Release Rate (*G*). This parameter (*G*) is estimated as the measure of the energy available for an increment of crack extension [25]. Using MD simulations, *G* is calculated from a cracked nano-crystal which is monotonically loaded until breaking [15,18,22,26]. However, the fracture toughness is established modeling a nano-crystal with a specific crack length and geometry that change from one investigation to other, therefore a fracture toughness that depends on the crack size is obtained, instead of a unique value that only depends on the material properties. The *J*-integral (*J*) is also an energy method that is extensively used to measure the fracture toughness [27,28,29]. Nevertheless, as other energy parameters, *J* lacks accuracy when irreversibility such as dislocations appear in the MD simulation [30]. In order to calculate *J*, Hardy’s approach is frequently implemented to estimate the local stress for atomistic systems as proposed by Zimmerman and Jones [31]. As in other atomistic investigations, Zimmerman and Jones estimate *J* modeling a nano-crystal with a unique initial crack length arbitrarily selected. Another method that is also widely utilized to estimate the fracture toughness is the crack tip opening displacement (CTOD). This FM parameter (CTOD) has been implemented by Thaulow et al. [32] to estimate the fracture toughness for silicon at the atomic scale. In addition, Thaulow et al. propose a method to estimate the plastic zone at the crack tip and the CTOD from MD simulations. In other research, Skogsrud and Thaulow perform atomistic modeling for a bbc-Fe cantilever beam at 500 K, 800 K and 1200 K, and estimate the fracture toughness using CTOD, but also using a unique and arbitrary initial crack length [16]. Skogsrud and Thaulow obtain a fracture toughness two times larger than values reported in the literature for the same material. In other investigation, Skogsrud and Thaulow [33] show that different crystallographic orientations in the same cantilever beam affect the fracture behavior, varying from ductile fracture for $\left(10\bar{1}\right)\left[101\right]$ crack system to brittle for $\left(100\right)\left[0\bar{1}1\right]$. Additionally, Skogsrud and Thaulow report that CTOD stops growing when dislocations are emitted from the crack tip during the MD simulation [33].

In the present work, based on MD simulations the fracture toughness $K_{c}$ is estimated for a single-crystal of Al by means of four different FM methods. The first method is the classical Irwing’s formulation for *K*. The second approach is a methodology proposed by Thaulow et al. [32]. The third method is based on a relationship between CTOD and the stress of the system. Lastly, *G* is estimated to calculate $K_{c}$ using a relationship between *G* and *K*. In order to establish an independent $K_{c}$ on the crack length using the four aforementioned FM methods, a methodology is proposed based on the analysis of cracked single-crystals where the crack length is varied. The proposed methodology is validated using published experimental data for graphene and data from MD simulations for diamond. The obtained $K_{c}$ for Al single-crystal is compare with other nanomaterials that present similar microstructures. Finally, the results are discussed and a comparison of the FM methods is carried out.

## 2. Methodology

### 2.1. Molecular Dynamics Simulation

**Figure 1:**
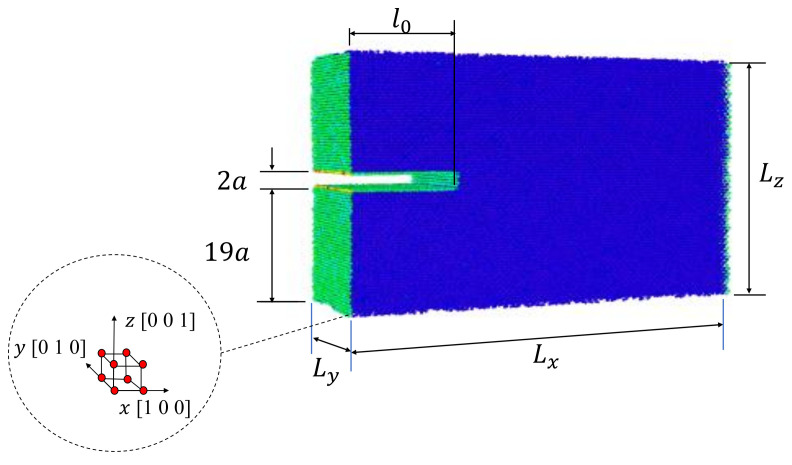
Single-crystal model geometry.

In this study, MD simulations are carried out using the code Large-scale Atomic/ Molecular Massively Parallel Simulator (LAMMPS) [34]. Single-crystals of Al with four different initial crack lengths are modeled. For each crystal, a uniaxial tensile test under controlled displacement is simulated. Initially, an isobaric-isothermal ensemble (NPT) is implemented to equilibrate the atomistic system using 20,000 steps. A deformation rate of 0.01% is applied to the single-crystal models in *z*-direction for 10,000 steps. The atomistic systems are relaxed using 20,000 steps (after every step deformation of 0.01%). A strain rate of $1\times 10^{-4}$ /ps and time-step of 0.001 ps are selected. The described process (deforming-relaxing) is repeated until fracture of the single-crystal. The embedded atom method (EAM) proposed by Mendelev et al. [35] is implemented in order to study the fracture process. EAM potential has been used to investigate crack propagation in Al crystals by several authors [21,30,36,37,38,39,40,41,42]. All simulations are carried out at 300 K and 1.01 bar using a Nose/Hoover thermostat and barostat. During the deformation process, free surface condition is established in the *x*-direction and periodic conditions in the others. Approximately 200,000 atoms conformed the atomistic system with dimension of 24×8×16 nm${}^{3}$ (Figure 1), with a lattice parameter a=0.405 nm for aluminum and initial crack lengths $l_{0}=5a,10a,15a,20a$. Virial stress tensor is utilized to compute the global stress as follow [43]:

(1) $$\sigma_{ij}=\frac{1}{V}\sum_{m=1}^{N}\left(\frac{1}{2}\sum_{m\neq n}^{N}F_{mn}^{i}r_{mn}^{j}-s_{m}v_{m}^{i}v_{m}^{j}\right)$$

where $\sigma_{ij}$ is the virial stress tensor, *V* is the atomistic system volume, $F_{mn}^{i}$ is the force vector between particle *m* and particle *n*, $r_{mn}^{j}$ is the distance vector between particle *m* and particle *n*, $s_{m}$ is the mass of the atom *m* and $v_{m}^{i}$ is the velocity vector of the atom *m*. The volume of the atoms is obtained using voro++ [44]. OVITO [45] is used to calculate the distance among atoms at the crack tip in order to estimate the CTOD. Using the dislocation extraction algorithm (DXA), the dislocations were analyzed [46].

### 2.2. Stress Intensity Factor Estimation

The stress intensity factor *K* is firstly estimated using the equation proposed by Irwin, as follows [47]:

(2) $$K_{I,1}=f\sigma \sqrt{\pi l}$$

where σ is the global stress, *l* is the crack length and *f* is the geometry factor for an edge crack, which is given by [47],

(3) $$f=0.265{\left(1-\alpha \right)}^{4}+\frac{0.857+0.265\alpha }{{\left(1-\alpha \right)}^{3/2}}$$

where α=l/60a. The same approach has been previously used to estimate $K_{c}$ from MD simulations in several publications [18,19,32,48].

**Figure 2:**
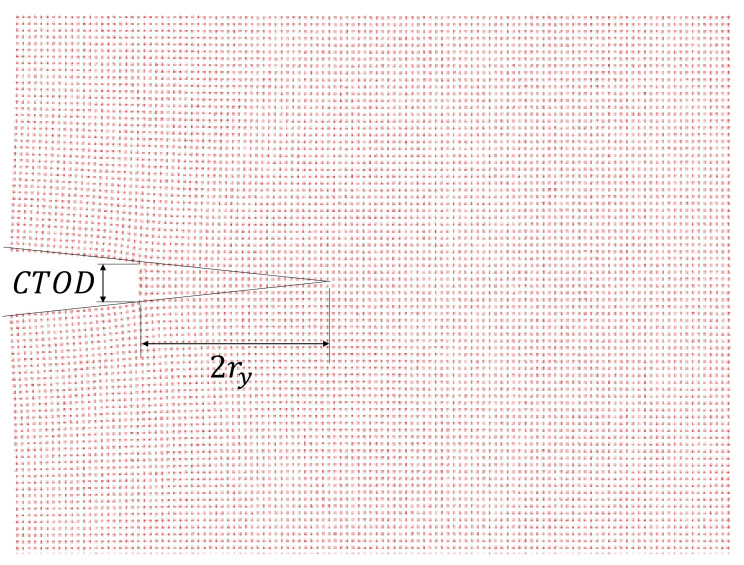
Crack tip opening displacement (CTOD) and plastic zone radius for a single-crystal Al with $l_{0}=10a$.

**Figure 3:**
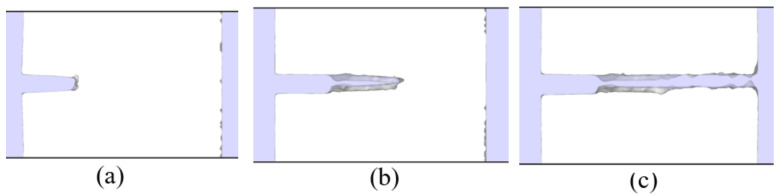
Dislocation analysis before and after starting the crack propagation (**a**) $\varepsilon_{zz}=6.7\%$ (**b**) $\varepsilon_{zz}=6.8\%$ and (**c**) $\varepsilon_{zz}=6.9\%$ for $l_{0}=15a$.

Another approach considered in this research is an equation implemented by Thaulow [32] to estimate *K* for atomistic cracked elements. In this equation, *K* is estimated based on the CTOD and the plastic zone size at the crack tip, as follows:

(4) $$K_{I,2}=\frac{E\sqrt{2\pi }}{8}\frac{CTOD}{\sqrt{r_{y}}}$$

where the Young’s modulus is E=60.58 GPa for an Al single-crystal [30,49] and $r_{y}$ is the plastic zone radius, which is estimated as proposed by Thaulow in [32] (Figure 2). At the atomistic scale, some researchers relate the dislocation emission region with the plastic zone size [50]. These dislocation emissions are considered as a source of energy release before crack propagation [51], however dislocation emissions are not observed during the simulations of the cracked single-crystals (Figure 3).

Another equation to estimate *K* from CTOD is given in [25], and it is defined by:

(5) $$K_{I,3}=\sqrt{\frac{\pi \cdot E\cdot CTOD\cdot \sigma }{4\cdot (1-\nu )}}$$

where the Poisson’s ratio is ν=0.36 for an Al single-crystal [30]. This approach has been implemented previously in [18].

**Figure 4:**
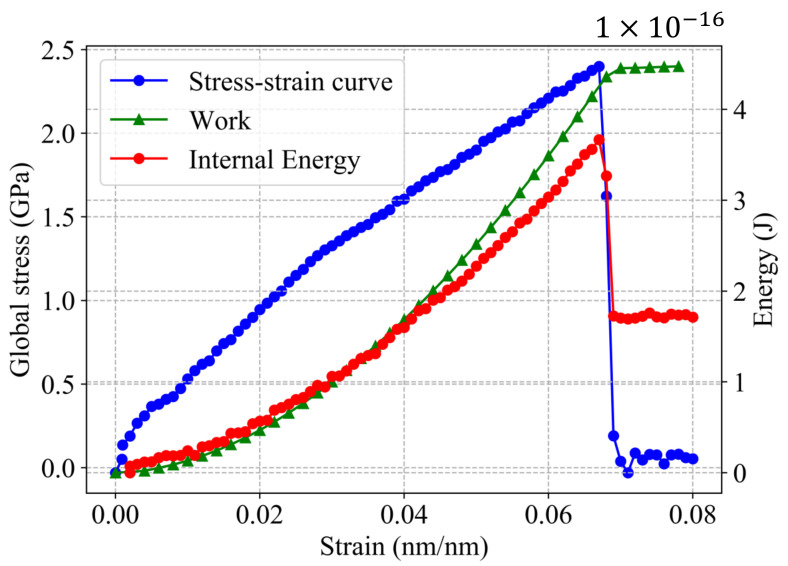
Stress–strain curve and energy behavior during deformation process in a single-crystal Al with $l_{0}=15a$.

Finally, *K* was also computed based on *G* using the Equation [25]:

(6) $$K_{I,4}=\sqrt{\frac{E}{1-\nu^{2}}\cdot G}$$

where *G* is estimated as the dissipated energy during the fracture per unit of new crack area created. *G* is calculated as follow:

(7) $$G=-\frac{\Delta (U-W)}{\Delta A}$$

where *U* is the potential energy of the system, ΔA is the new crack surface created due to fracture and *W* is the work applied during the tensile test before the fracture ($\varepsilon_{f}=6.7\%$, Figure 4). The initial work and potential energy are assumed to be negligible. Thus, *W* is estimated from the stress–strain curve (Figure 4) as follows [47]:

(8) $$W=V\int_{0}^{\varepsilon_{f}}\sigma d\varepsilon$$

where *V* is the atomistic system volume. Jung et al. has used this method to estimate *G* from MD simulation [22].

## 3. Results and Discussion

### 3.1. Fracture Behavior

By means of dislocation extraction algorithm (DXA) [46], a dislocation analysis is carried out. The DXA identifies the dislocation lines in a nano-crystal in case that dislocations appear during the simulation. Based on this analysis, dislocation emissions are not evidenced during the fracture process (Figure 3), therefore brittle behavior is obtained in the MD simulations.

### 3.2. Stress Intensity Factor Assessment

The data used to calculate *K* by means of the different approaches, viz. Equations (2), (4), (5) and (6), are summarized in Table 1. These values are computed just before the fracture. The *K* values are presented in Table 2.

### 3.3. Fracture Toughness for Al Single-Crystal

In order to establish a $K_{c}$ value that is independent on the crack length, an equivalent stress $\sigma_{eq}$ is computed based on the $K_{I}$ in Table 2. $\sigma_{eq}$ is calculated as:

(9) $$\sigma_{eq}=\frac{K_{I}}{f\cdot \sqrt{\pi \cdot l_{0}}}$$

**Figure 5:**
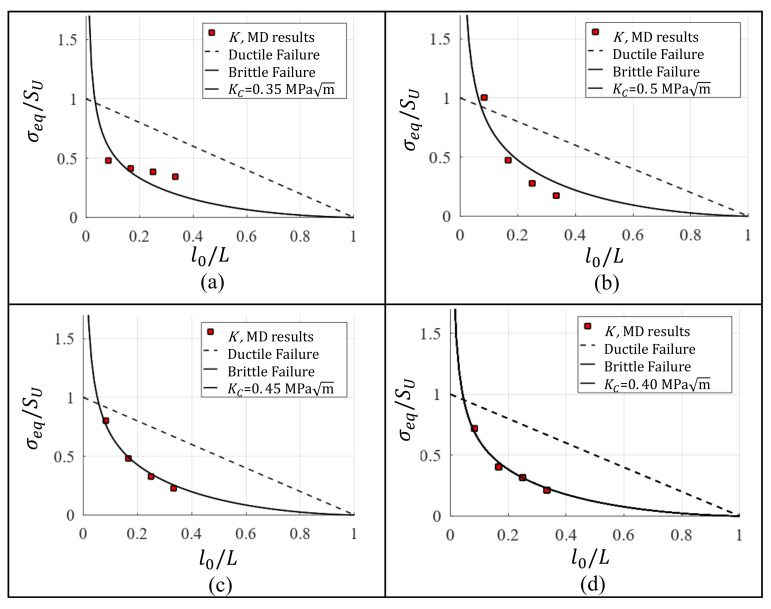
Fracture toughness estimation for Al single-crystal based on (**a**) *K*, (**b**) CTOD-plastic-zone, (**c**) CTOD−σ and (**d**) *G*.

A dimensionless value is obtained dividing $\sigma_{eq}$ by $S_{U}$ for a single-crystal of Al without defects, viz., $S_{U}=6.182$ GPa [30]. Table 3 summarizes $\sigma_{eq}/S_{U}$, which is computed using the data from Table 2. $\sigma_{eq}/S_{U}$ from Table 3 vs. $l_{0}/L$ (where L=60a) was plotted in Figure 5. Using the least squares method a curve based on Equation (9) is fitted to the data, thus $K_{c}$ is the $K_{I}$ that yields the curve with best fit (Figure 5). $K_{c}$ values estimated by means of the different approaches are presented in Table 4. The residual sum of squares (RSS) is implemented to determine the accuracy of the methods to predict $K_{c}$, and the results are summarized in Table 5. The obtained lowest error is 0.00252, which corresponds to *G* method, however CTOD−σ obtained almost the same error, viz., 0.00260. Additionally, $K_{c}$ is in the same order of magnitude reported by Chandra [36]. However, the fracture toughness reported by Chandra is determined modeling a single-crystal with a unique initial crack length that is arbitrarily chosen.

### 3.4. Fracture Toughness for Graphene and Diamond

**Figure 6:**
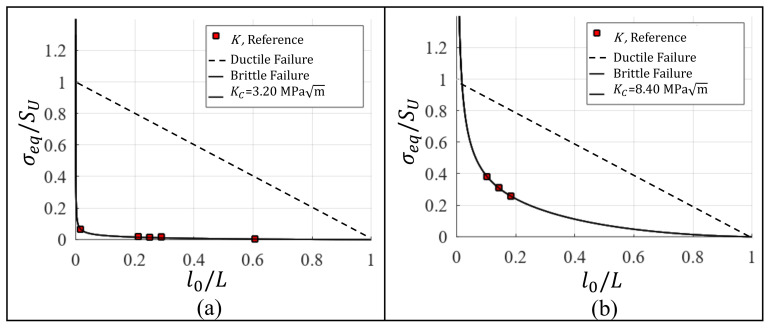
Fracture toughness estimation for (**a**) graphene and (**b**) diamond.

The same methodology that is applied in Section 3.3 to establish $K_{c}$ for Al single-crystal is also used to estimate the $K_{c}$ for other two nanomaterials by analyzing data from published scientific articles. Table 6 presents the $K_{c}$ for cracked specimens of graphene and diamond with different crack lengths. The data for graphene are experimentally obtained in [52] and for the diamond by means of MD simulations in [18]. Using data from Table 6, $\sigma_{eq}$ is calculated by Equation (9) and normalized dividing $\sigma_{eq}$ by $S_{U}$, where $S_{U}=130$ GPa for graphene [53] and $S_{U}=241.2$ GPa for diamond [18]. Figure 6a,b show $\sigma_{eq}/S_{U}$ vs. $l_{0}/L$ and the respective adjusted curves to obtain the fracture toughness. The $K_{c}$ that is obtained for graphene and diamond based on the proposed methodology are shown in Table 7 with the respective errors which are calculated by means of the residual sum of squares.

### 3.5. Fracture Toughness Comparison

**Figure 7:**
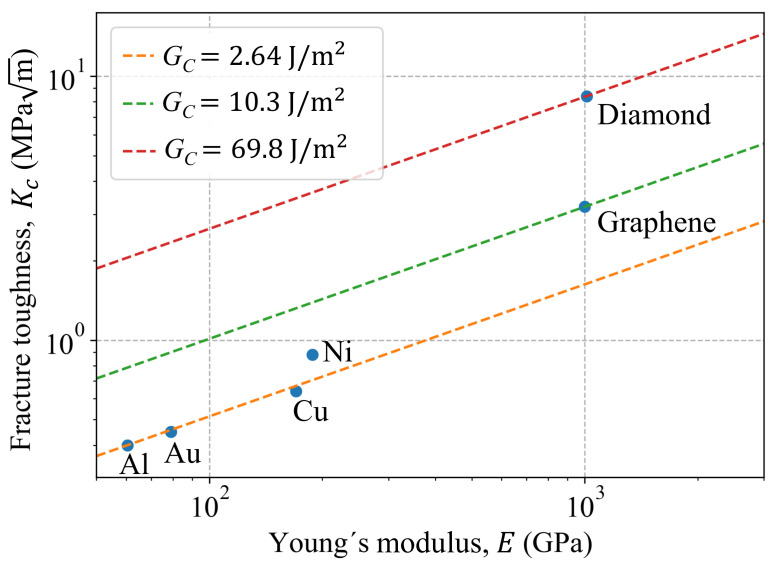
Fracture toughness comparison for some nanomaterials.

In order to compare the fracture toughness of the Al single-crystal to other nanomaterials with similar microstructures, $K_{c}$ vs. *E* is plotted in Figure 7. The different nanomaterials and their properties are summarized in Table 8, where $G_{c}$ is estimated as follows:

(10) $$G_{c}=K_{c}^{2}/E$$

The fracture toughness for graphene and diamond are also plotted in the same figure as reference values. As seen in Figure 7, materials with FCC microstructures show similar fracture behavior, viz.: Al, Au, Cu and Ni. It is important to point out that $K_{c}$ for Au, Cu and Ni are estimated in [54,55,56,57] based on modeling nano-crystals with a unique and arbitrary selected crack length.

## 4. Conclusions

In this research, the fracture toughness for Al single-crystals has been assessed successfully by means of four different approaches. The fracture toughness for graphene and diamond has been also estimated using published data by other researchers. Finally, $K_{c}$ for the Al single-crystal has been compared with nanomaterials that have microstructures similar to the Al. From the results, it is concluded:

- The fracture behavior observed in the MD simulations is in accordance with observations reported by other researchers in their investigations on similar Al crystals.
- Despite that Al is considered a ductile material at 300 K, a brittle fracture behavior is observed for single-crystals in the MD simulations.
- The methodology that is proposed in this research provides a suitable method to obtain a fracture toughness value that is independent on the crack length.
- The parameters *G* and CTOD−σ yield a good accuracy to predict the fracture of single-crystals.
- Dislocations are not observed during the simulations. Therefore, it is not possible to compare the plastic zone estimations with the dislocation emission zone.

## Figures and Tables

**Table 1:** Data from the molecular dynamics (MD) simulations to estimate *K*.

$l_{0}$ (m)	α	*f*	σ (GPa)	CTOD (m)	$r_{y}$ (m)	*W* (fJ)	*U* (fJ)	ΔA (m${}^{2}$)
2.03 $\times \;10^{-9}$	8.33 $\times \;10^{-2}$	1.19	2.96	0.137 $\times \;10^{-8}$	1.95 $\times \;10^{-9}$	0.62	0.21	1.61 $\times \;10^{-16}$
4.05 $\times \;10^{-9}$	1.67 $\times \;10^{-1}$	1.31	2.56	0.141 $\times \;10^{-8}$	3.81 $\times \;10^{-9}$	0.48	0.17	1.58 $\times \;10^{-16}$
6.08 $\times \;10^{-9}$	2.50 $\times \;10^{-1}$	1.51	2.38	0.140 $\times \;10^{-8}$	5.51 $\times \;10^{-9}$	0.44	0.13	1.34 $\times \;10^{-16}$
8.10 $\times \;10^{-9}$	3.33 $\times \;10^{-1}$	1.79	2.13	0.143 $\times \;10^{-8}$	7.79 $\times \;10^{-9}$	0.41	0.15	1.28 $\times \;10^{-16}$

**Table 2:** Stress intensity factor just before fracture.

$l_{0}$ (m)	$K_{I,1}$ (MPa$\cdot \sqrt{m}$)	$K_{I,2}$ (MPa$\cdot \sqrt{m}$)	$K_{I,3}$ (MPa$\cdot \sqrt{m}$)	$K_{I,4}$ (MPa$\cdot \sqrt{m}$)
2.03 $\times \;10^{-9}$	0.281	0.588	0.471	0.421
4.05 $\times \;10^{-9}$	0.378	0.433	0.443	0.369
6.08 $\times \;10^{-9}$	0.494	0.357	0.426	0.405
8.10 $\times \;10^{-9}$	0.606	0.307	0.407	0.375

**Table 3:** Dimensionless equivalent stress.

$l_{0}$ (m)	$\sigma_{\mathrm{eq}}/S_{U}\left(K_{I,1}\right)$	$\sigma_{\mathrm{eq}}/S_{U}\left(K_{I,2}\right)$	$\sigma_{\mathrm{eq}}/S_{U}\left(K_{I,3}\right)$	$\sigma_{\mathrm{eq}}/S_{U}\left(K_{I,4}\right)$
2.03 $\times \;10^{-9}$	0.48	1.00	0.80	0.72
4.05 $\times \;10^{-9}$	0.41	0.47	0.48	0.40
6.08 $\times \;10^{-9}$	0.38	0.28	0.33	0.31
8.10 $\times \;10^{-9}$	0.34	0.17	0.23	0.21

**Table 4:** Fracture toughness for Al single-crystal.

$K_{c}-K$ (MPa$\cdot \sqrt{m})$	$K_{c}-\mathrm{CTOD}$-pz (MPa$\cdot \sqrt{m})$	$K_{c}-\mathrm{CTOD}-\sigma$ (MPa$\cdot \sqrt{m})$	$K_{c}-G$ (MPa$\cdot \sqrt{m})$
0.35	0.50	0.45	0.40

**Table 5:** Residual sum of squares for the different methods.

*K*	CTOD-pz	CTOD−σ	*G*
0.04871	0.05234	0.00260	0.00252

**Table 6:** Fracture toughness and crack length for graphene and diamond.

Material	Crack Length (nm)	$K_{c}$ (MPa$\cdot \sqrt{m})$	Reference
Graphene	33	3.1	[52]
	438	4.1	
	518	3.7	
	600	4.9	
	1256	4.1	
Diamond	1.785	8.360	[18]
	2.499	8.463	
	3.213	8.405	

**Table 7:** Fracture toughness for graphene and diamond and the respective error.

Material	$K_{c}$ (MPa$\cdot \sqrt{m})$	*E* (GPa)	$G_{c}$ (J/m${}^{2}$)	Error
Graphene	3.20	1000	10.3	1.03 $\times 10^{-4}$
Diamond	8.40	1011.5	69.8	8.65 $\times 10^{-6}$

**Table 8:** FM properties for different nanomaterials.

Material	$K_{c}$ (MPa$\cdot \sqrt{m})$	*E* (GPa)	$G_{c}$ (J/m${}^{2}$)	Reference
Au	0.45	79	2.56	[54,55]
Cu	0.64	169.9	2.43	[56]
Ni	0.88	188	4.12	[57]

## Data Availability

Data is contained within the present article.
